# Complete Genome Analysis of Influenza A(H1N1) Viruses Isolated in Kerala, India

**DOI:** 10.1128/MRA.00062-20

**Published:** 2020-03-19

**Authors:** Raji Prasad, Vishnu VikramanThampi Mohanakumari, Remya Vasanthi Sasi, Radhakrishnan Nair, Sara Jones, Madhavan Radhakrishna Pillai

**Affiliations:** a Pathogen Biology Program, Rajiv Gandhi Centre for Biotechnology, Thiruvananthapuram, Kerala, India; b Laboratory Medicine and Molecular Diagnostic, Rajiv Gandhi Centre for Biotechnology, Thiruvananthapuram, Kerala, India; Queens College

## Abstract

Here, we report the complete sequence of four influenza A(H1N1) virus samples isolated from cases that occurred during the 2017 epidemic season in Kerala in South India. Sequence analysis showed mutations that differentiate this strain from the reference strain A/California/07/2009 virus.

## ANNOUNCEMENT

Influenza pandemic continues to be a major concern throughout the world. During the first half of 2017, India saw an increase in morbidity and mortality rates caused by influenza A virus (1, 2). There has been a drastic change in the seasonality of occurrence, with major outbreaks in the dry, hot summer months (3–5). Adequate surveillance data are not available in the public domain to make detailed assessments of the trends in circulation. The genetic variation which made this possible also remains to be elucidated.

One hundred seventy-nine nasopharyngeal swabs were collected from hospitalized patients who exhibited a broad spectrum of clinical syndromes ranging from upper respiratory illness and pneumonia to bronchitis from January to March 2017. Ethical clearance for this study was provided by the Institutional Human Ethics Committee of Rajiv Gandhi Centre for Biotechnology (no. IHEC/1/2013/01). Sample processing was carried out as per WHO guidelines (6). Viral RNA was extracted using the MN viral RNA isolation kit (Macherey-Nagel GmbH & Co. KG, Germany). The *in vitro* qualitative detection of novel H1N1 2009 virus from respiratory specimens was performed using the Real Star influenza reverse transcription-PCR (RT-PCR) kit 2.0 (Altona GmbH). Out of 179 samples, 25 tested positive for influenza A(H1N1) virus. We report the complete genome sequence of four of these positive samples. All eight gene segments were amplified directly by RT-PCR using the recommended WHO whole-genome overlapping sequencing primers (http://www.who.Int/csr/resources/publications/swineflu/GenomePrimers_20090512.pdf) in four to six fragments of 400 to 600 bp with 100-bp overlap to obtain 4-fold sequence coverage. Samples were sequenced on an ABI 3500 DNA analyzer. Data were analyzed using sequence scanner software version 2 and aligned using Bio Edit version 7.2.5.

All gene segments were aligned against the reference strain A/California/07/2009, along with globally circulating H1N1 strains. Sequence analysis revealed that the 2017 circulating strains from Kerala acquired several mutations over time. We have previously reported two new mutations, S181T and I312V, in the HA gene, which could lead to altered glycan specificity (7). None of the isolates harbored the neuraminidase drug resistance mutations in the NA gene. Similarly, we observed the following point mutations in genes of all four sequenced samples, along with mutations reported during the pandemic season (8, 9): R54K, M66I, D195N, R293K, R299K, V344M, I354L, S453T, and V731I in polymerase basic 2 (PB2); F94L, G154D, I397M, K430R, and I435T in polymerase basic 1 (PB1); X18E, V100I, P224S, N321K, I330V, and R362K in polymerase acidic (PA); D2E, E55K, L90I, I123V, E125D, K131E, and N205S in nonstructural protein (NS); A22T, V100I, M105T, X114E, L122Q, and S498N in nucleoprotein (NP); V80I, M192V, Q208K, and K230R in matrix (M); A13T, S91R, P100S, S101N, D114N, S179N, K180Q, S181T, S202T, A212T, S220T, I233T, A273T, K300E, I312V, I338V, E391K, S468N, E516K, and V544I in hemagglutinin (HA); and V13I, I34V, L40I, N44S, G77R, I188T, N200S, V241I, N248D, V264I, N270K, I314M, I321V, N369K, N386K, K432E, and N449D in neuraminidase (NA).

### Data availability.

The complete genome sequences of influenza A(H1N1) viruses isolated in Kerala, India, in 2017 have been deposited in GenBank under the accession numbers listed in Table 1.

**TABLE 1:** Accession numbers of influenza A(H1N1) gene sequences deposited in GenBank

Isolate	GenBank accession no. for gene:							
	PB2	PB1	PA	HA	M	NP	NA	NS
RGCB_2108	MN744387	MN744386	MN744385	MF510838	MN744383	MN744388	MF510842	MN744384
RGCB_2302	MN744410	MN744409	MN744408	MF510841	MN744407	MN744411	MF510845	MN744412
RGCB_2247	MN744391	MN744390	MN744389	MF510839	MN744392	MN744393	MF510844	MN744394
RGCB_2263	MN744403	MN744402	MN744401	MF510840	MN744404	MN744406	MN960321	MN744405
